# The effect of immunomodulatory drugs on aortic stenosis: a Mendelian randomisation analysis

**DOI:** 10.1038/s41598-023-44387-x

**Published:** 2023-11-01

**Authors:** Jonathan L. Ciofani, Daniel Han, Milad Nazarzadeh, Usaid K. Allahwala, Giovanni Luigi De Maria, Adrian P. Banning, Ravinay Bhindi, Kazem Rahimi

**Affiliations:** 1 Department of Epidemiology and Biostatistics, School of Public Health, Imperial College London, London, UK; 2 Department of Cardiology, Royal North Shore Hospital, Sydney, Australia; 3 Sydney Medical School, The University of Sydney, Sydney, Australia; 4 Medical Research Council Laboratory of Molecular Biology, Cambridge, UK; 5 Department of Physiology, Development and Neuroscience, University of Cambridge, Cambridge, UK; 6 School of Mathematics and Statistics, University of New South Wales, Sydney, Australia; 7 Deep Medicine, Oxford Martin School, University of Oxford, Oxford, UK; 8 Nuffield Department of Women’s and Reproductive Health, Medical Science Division, University of Oxford, Oxford, OX1 2BQ UK; 9 Oxford University Hospitals NHS Foundation Trust, Oxford, UK; 10 National Institute for Health Research Oxford Biomedical Research Centre, Oxford University Hospitals NHS Foundation Trust, Oxford, UK

**Keywords:** Genetics, Immunology, Cardiology

## Abstract

There are currently no approved pharmacological treatment options for aortic stenosis (AS), and there are limited identified drug targets for this chronic condition. It remains unclear whether inflammation plays a role in AS pathogenesis and whether immunomodulation could become a therapeutic target. We evaluated the potentially causal association between inflammation and AS by investigating the genetically proxied effects of tocilizumab (IL6 receptor, IL6R, inhibitor), canakinumab (IL1β inhibitor) and colchicine (β-tubulin inhibitor) through a Mendelian randomisation (MR) approach. Genetic proxies for these drugs were identified as single nucleotide polymorphisms (SNPs) in the gene, enhancer or promoter regions of *IL6R*, *IL1β* or β-tubulin gene isoforms, respectively, that were significantly associated with serum C-reactive protein (CRP) in a large European genome-wide association study (GWAS; 575,531 participants). These were paired with summary statistics from a large GWAS of AS in European patients (653,867 participants) to then perform primary inverse-variance weighted random effect and sensitivity MR analyses for each exposure. This analysis showed that genetically proxied tocilizumab was associated with reduced risk of AS (OR 0.56, 95% CI 0.45–0.70 per unit decrease in genetically predicted log-transformed CRP). Genetically proxied canakinumab was not associated with risk of AS (OR 0.80, 95% CI 0.51–1.26), and only one suitable SNP was identified to proxy the effect of colchicine (OR 34.37, 95% CI 1.99–592.89). The finding that genetically proxied tocilizumab was associated with reduced risk of AS is concordant with an inflammatory hypothesis of AS pathogenesis. Inhibition of IL6R may be a promising therapeutic target for AS management.

## Introduction

The prevalence of aortic stenosis (AS) is increasing with the ageing population. Approximately 12% of individuals over age 75 years are diagnosed with AS, of whom over a quarter have severe AS^1^. If left untreated, severe AS can lead to up to 50% mortality within two years of symptom onset^2^. Transcatheter and surgical valve replacement are currently the mainstays of treatment^3,4^, with a limited role for medical therapies. The 2020 American guidelines for management of valvular heart disease recognised a role for treatment of dyslipidemia and hypertension, particularly with angiotensin-converting enzyme inhibitors or angiotensin receptor blockers^3,5–8^. Similarly the 2021 European guidelines advised treatment of coexistent hypertension, although these guidelines also importantly note that medical therapies have not yet been shown to alter the natural history of AS^4^. There is consequently a need to better understand the causal mechanisms underlying AS to facilitate targeted preventative and therapeutic medications.

Several modalities of evidence suggest that inflammation plays a role in the development of AS. Immunohistochemical analysis of surgical and autopsy aortic valves has demonstrated elevated expression of inflammatory cell adhesion molecules and cytokines, and increased inflammatory cell infiltration in valves with AS compared to normal valves^9–12^. Positron emission tomography concordantly demonstrates increased tracer uptake in patients with AS versus normal valves^13^. A genome-wide association study (GWAS) meta-analysis further identified the *IL6* locus as significantly associated with AS^14^ and observational studies have demonstrated an association between increased IL6 protein levels and AS^15,16^. However, whether inflammation is a cause, consequence or correlate of AS remains unclear. Several studies have shown similarities in the pathogenesis between AS and coronary atherosclerosis, and several randomised trials have demonstrated the effectiveness of the anti-inflammatory medications canakinumab^17^ and colchicine^18,19^ for coronary heart disease. It is therefore hypothesised that immunomodulatory drugs, such as tocilizumab (IL6 receptor, IL6R, inhibitor), canakinumab (IL1β inhibitor) or colchicine (β-tubulin inhibitor) may prove effective for the management of AS.

Mendelian randomisation (MR) offers an opportunity to investigate the potentially causal relationship between risk factors of interest, such as inflammation, and outcomes, such as AS^5,20^. This approach has previously been used to demonstrate significant associations between both raised systolic blood pressure and low-density lipoprotein cholesterol with risk of not only coronary disease^21^ but also AS^5,7^. MR relies on the premise that a proportion of an individual’s phenotype is determined by genetic polymorphisms that are randomly inherited at birth. The random inheritance of phenotype-determining genetic polymorphisms is analogous to assignment to a treatment group in a randomised control trial^22^. This randomisation process can overcome several issues with observational studies including reverse causation and confounding, and may therefore facilitate causal inferences when clinical trials are unavailable.

The MR approach has recently been extended to estimate the effects of immunomodulation on important clinical outcomes. C-reactive protein (CRP) is a well-established marker of systemic inflammation and is consequently a useful clinical indicator to evaluate the effectiveness of anti-inflammatory treatments. Several studies have proxied the effects of inhibiting IL6 pathway signalling by identifying single-nucleotide polymorphisms (SNPs) that are both significantly associated with CRP levels and located in or near to the *IL6R* gene. This approach has been used in MR analyses to demonstrate that genetically proxied IL6R inhibition is significantly associated with reduced risk of coronary artery disease and ischemic stroke^23–25^. Moreover, it has previously been shown that genetically proxied IL6R inhibition, estimated using a SNP in the *IL6R* gene, is associated with a similar biochemical effect as administering tocilizumab^26^. Of note, however, the potential effect of immunomodulation on risk of AS has not yet been studied.

The present study uses the MR approach with genetic proxies of the immunomodulatory drugs tocilizumab, canakinumab and colchicine to investigate the potentially causal role of inflammation in AS pathogenesis and to predict whether administration of these drugs would be likely to reduce the risk of developing AS.

## Methods

### Study design

**Figure 1:**
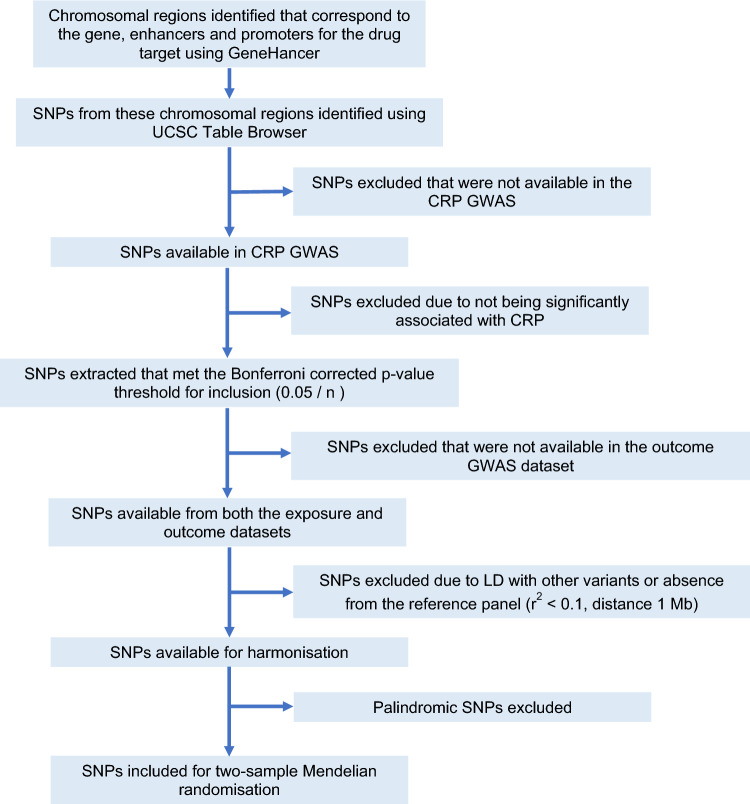
Flow chart of SNP selection process for drug proxies. Further details for each specific drug proxy are available in Supplementary Fig. 1. *CRP* C-reactive protein, *SNP* single nucleotide polymorphism, *GWAS* genome wide association study, *n* the cumulative number of SNPs extracted from the gene, enhancer and promoter regions for a given drug proxy, *LD* linkage disequilibrium.

This study uses a two-sample MR approach with summary data from published studies. Three major anti-inflammatory drugs were considered in this study: tocilizumab, canakinumab and colchicine. Canakinumab and colchicine were selected based on recent trials demonstrating effectiveness for coronary disease^17–19^. Tocilizumab was selected based on preliminary evidence suggesting a role of IL6 in AS^14–16^. The method for identifying genetic proxies and estimating their anti-inflammatory effect was adapted from previously published approaches^23,27^ and is summarised in Fig. 1. Ethics approval and consent were obtained by the original studies detailed below^28–31^.

### Data sources

Genes encoding the protein targets of the anti-inflammatory drugs under investigation were identified using the DrugBank database^32^. This included *IL1β* for canakinumab, *IL6R* for tocilizumab and *TUBB, TUBB2A, TUBB2B, TUBB3, TUBB4A, TUBB4B, TUBB6, TUBB1, TUBB8* and *TUBB8B* for colchicine. The gene, promoter and enhancer regions for each of these genes were identified using the GeneCards platform^33^ (Supplementary Tables 1–3). SNPs in these regions were determined using the UCSC online platform^34^. Of these, SNPs that may proxy the anti-inflammatory effects of the aforementioned drugs were identified based on association with natural log-transformed peripheral C-reactive protein (CRP) levels (mg/L) in a genome-wide association study (GWAS) meta-analysis of UK Biobank and Cohorts for Heart and Aging Research in Genomic Epidemiology (CHARGE) study participants^28^ at a Bonferroni-corrected p value threshold (p < 0.05/n, where n is the cumulative number of SNPs extracted from the gene, enhancer and promoter regions for a given drug proxy). SNPs were clumped to maximise sensitivity using a linkage disequilibrium threshold of *r*^2^ < 0.1 and 1 Mb distance cut-off using the 1000 genomes European reference panel. Palindromic SNPs were excluded. SNPs used to proxy each anti-inflammatory drug are presented in Supplementary Table 4. The genetic variant selection process is summarised in Fig. 1 and Supplementary Fig. 1.

Summary statistics for the association between the SNPs identified above and CRP levels were extracted from the GWAS meta-analysis of UK Biobank and CHARGE participants^28^. This is the largest GWAS on CRP to date and includes 575,531 participants of European descent. Further details are available in Supplementary Table 5.

Aortic stenosis was the primary outcome variable. Summary statistics were obtained from a meta-analysis of 10 European cohorts, including 13,765 cases and 640,102 controls^35^. A summary of the significant SNPs identified in this meta-analysis are reported in Supplementary Table 10. Complete description of each cohort and their case definition is available from the original study^35^.

### Statistical analyses

The primary MR analysis was an inverse variance weighted (IVW) random effect model, with results expressed as odds ratios (OR) on the outcome per unit decrease in genetically predicted natural log-transformed CRP level. For exposures with only a single suitable SNP identified, Wald ratios were calculated for the primary analysis.

To evaluate the robustness of findings, we performed several sensitivity analyses including weighted-median, weighted-mode, MR-PRESSO, MR-Egger, MR-Robust Adjusted Profile Score (MR-RAPS) and leave-one-out sensitivity analyses^22,36,37^. Each method makes different assumptions and thus concordance between methods provides confidence in the conclusion. The weighted-median approach assumes that at least half the instrumental variables are valid. Weighted-mode assumes that the most common causal effect is consistent with the true effect. MR-PRESSO consists of three steps: (1) the MR-PRESSO global test evaluates for horizontal pleiotropy; (2) calculation of the outlier-corrected causal estimate which corrects for horizonal pleiotropy that has been detected; and (3) the MR-PRESSO distortion test, which evaluates whether the causal estimate differs significantly after correction for outliers at p < 0.05 threshold^37^. MR-Egger uses the Instrument Strength Independent of Direct Effect assumption, which states that pleiotropic effects from the genetic variants to the outcome are independent of the association between genetic variants and exposure. Under this assumption, the intercept from MR-Egger analysis estimates the average pleiotropic effect of the genetic variants^38^. MR-RAPS is robust to idiosyncratic and systematic pleiotropy^36^. Leave-one-out sensitivity analysis involves removing a single variant from the analysis in turn then assessing the fluctuation in the estimate for possible outlier contribution. Outliers were further identified using Cook’s distance method and the above analyses re-performed using an outlier-excluded dataset. Heterogeneity of the genetic variants was assessed using scatter plots, funnel plots and applying Cochran’s Q-statistic.

Positive and negative controls were included. Rheumatoid arthritis (RA) was used as the positive control outcome, with summary statistics obtained from a large GWAS meta-analysis that included 22,686 RA cases and 288,644 controls (58,284 European and 253,008 East Asian ancestry)^29^. Osteoarthritis (OA) was used as the negative control outcome, with summary statistics obtained from a GWAS of European ancestry UK Biobank participants that included 10,083 cases and 40,425 controls^30^. Additionally, analyses were conducted for coronary artery disease (CAD) as the outcome to compare with the results for AS. These analyses used summary statistics from a large GWAS meta-analysis of 48 studies including 60,801 coronary artery disease cases and 123,504 controls^39^.

Statistical analyses were performed using R version 1.4.1106^40^.

## Results

There were 18, 9 and 1 suitable SNPs identified to proxy the effects of tocilizumab (IL6R antagonist), canakinumab (IL1β inhibitor) and colchicine (β-tubulin inhibitor), respectively (Supplementary Tables 4 and 6).

**Figure 2:**
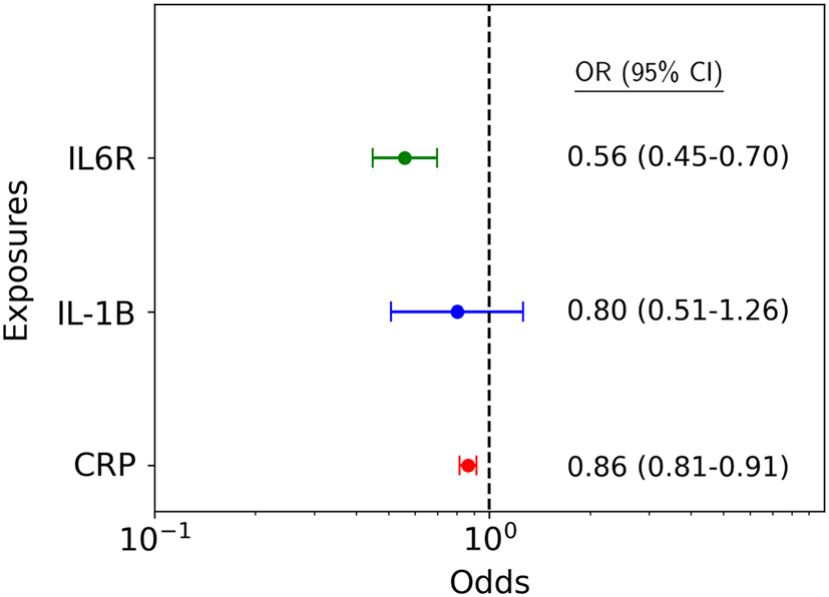
Mendelian randomisation inverse variance weighted estimates for the effect per unit decrease in exposure-mediated natural log transformed C-reactive protein (CRP) levels on risk of aortic stenosis. The exposures of interest included interleukin 6 receptor-mediated (IL6R), IL1β-mediated (IL1B) and overall genetically predicted CRP. *OR* odds ratio, *CI* confidence interval.

**Figure 3:**
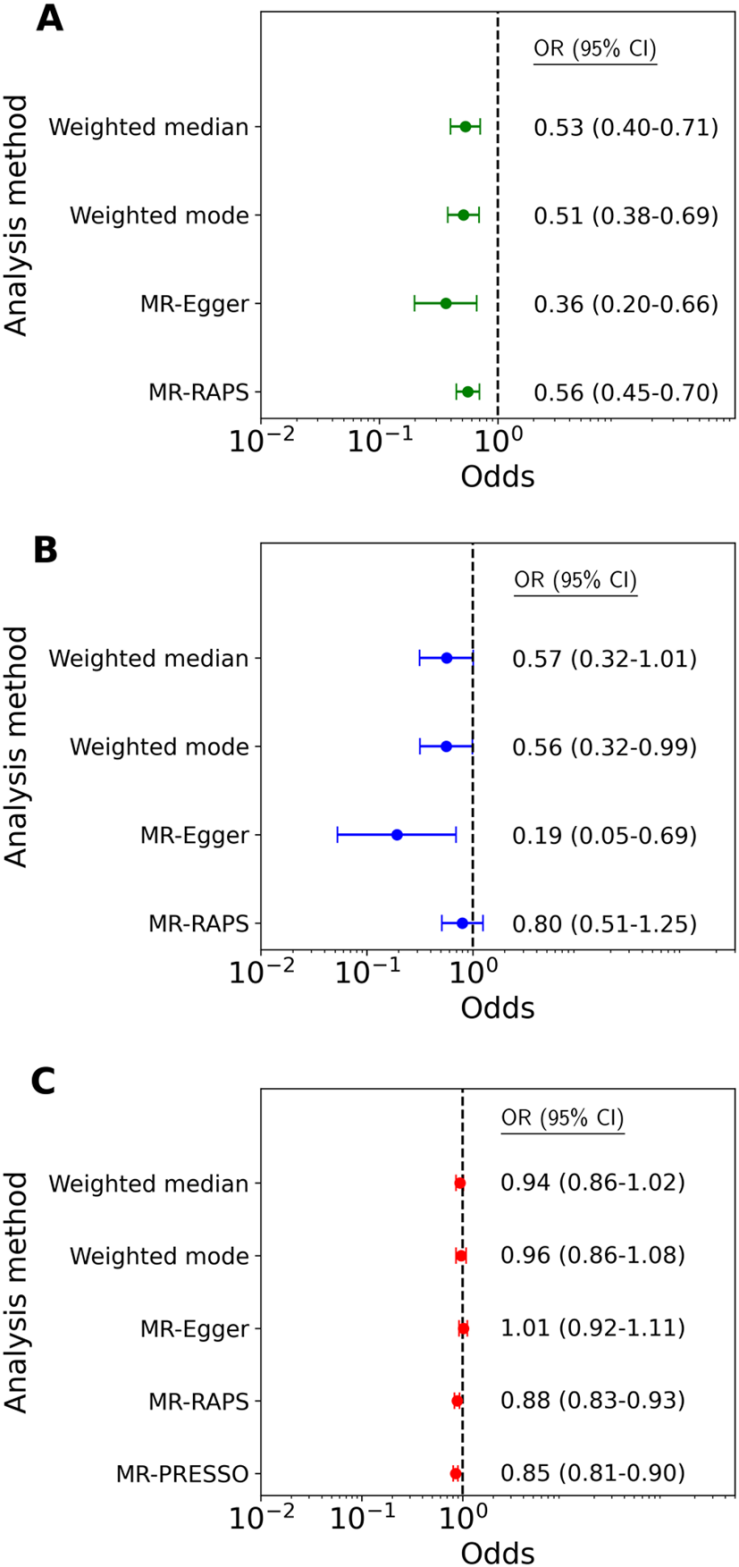
Sensitivity analyses for the effect per unit decrease in exposure-mediated natural log transformed C-reactive protein (CRP) levels on risk of aortic stenosis. The exposures of interest included: (**A**) interleukin 6 receptor-mediated (IL6R); (**B**) IL1β-mediated (IL1B); and (**C**) overall genetically predicted CRP. *OR* od ds ratio, *CI* confidence interval.

Genetically proxied inhibition of IL6R was significantly associated with reduced risk of AS on IVW primary analysis (OR 0.56, 95% CI 0.45–0.70) (Fig. 2). This was robust to all sensitivity analyses (weighted median OR 0.53, 95% CI 0.40–0.71; weighted mode OR 0.51, 95% CI 0.38–0.69; MR-RAPS OR 0.56, 95% CI 0.45–0.70; MR-Egger OR 0.36, 95% CI 0.20–0.66) (Fig. 3; Supplementary Table 6). Leave-one-out analyses are presented in Supplementary Fig. 2. The MR-Egger intercept was non-significant (Supplementary Table 7) and there were no significant outliers on MR-PRESSO analysis. There was no significant heterogeneity on IVW analysis (Q statistic 14.05, p = 0.66) (Supplementary Table 8). Scatter and funnel plots are presented in Supplementary Figs. 3 and 4. The IVW analysis was robust to exclusion of two outliers identified by Cook’s distance (OR 0.56, 95% CI 0.44–0.73), and MR sensitivity analyses demonstrated consistent results upon exclusion of outliers (Supplementary Figs. 5–8).

Genetically proxied inhibition of IL1β did not meet statistical significance for association with reduced risk of AS on IVW primary analysis (OR 0.80, 95% CI 0.51–1.26) (Fig. 2). The sensitivity analyses were largely consistent with this result (weighted median OR 0.57, 95% CI 0.32–1.01; weighted mode OR 0.56, 95% CI 0.32–0.99; MR-RAPS OR 0.80, 95% CI 0.51–1.25), with only the MR-Egger analysis reaching statistical significance (OR 0.19, 95% CI 0.05–0.69). The MR-Egger intercept was non-significant (Supplementary Table 7) and there were no significant outliers on MR-PRESSO analysis. There was no significant heterogeneity on IVW analysis (Q statistic 8.31, p = 0.40). Similarly neutral results were identified upon exclusion of two outlier SNPs identified by Cook’s distance (Supplementary Table 9).

Genetically proxied inhibition of β-tubulin was associated with increased risk of AS by Wald ratio analysis (OR 34.37, 95% CI 1.99–592.89). This was based on identification of a single suitable SNP and consequently sensitivity analyses could not be performed.

There was a significant association between genetically predicted lower CRP and AS on IVW primary analysis (OR 0.86, 95% CI 0.81–0.91) (Fig. 2; Supplementary Figs. 3 and 4). However this was not robust to sensitivity analyses by weighted median, weighted mode nor MR-Egger analysis (Supplementary Table 5). Significant pleiotropy was identified with an Egger intercept of 0.0060 (p < 0.0001) and significant heterogeneity was identified in the IVW analysis (Q statistic 1514.63, p < 0.0001).

The positive and negative controls corresponded to broadly expected results. Significant associations were identified between genetically proxied IL6R and IL1β mediated lower CRP levels and reduced risk of RA on primary IVW (IL6R: OR 0.57, 95% CI 0.39–0.82; IL1β: OR 0.49, 95% CI 0.30–0.79) and sensitivity analyses. There was no association identified between genetically proxied β-tubulin inhibition and RA on Wald ratio (OR 0.72, 95% CI 0.02–21.51). There was a significant association between genetically proxied reduced CRP and risk of RA on primary IVW analysis (OR 0.93, 95% CI 0.87–0.98) but this was not robust to sensitivity analyses (Table 1; Supplementary Tables 6–9).

**Table 1:** Mendelian randomisation inverse variance weighted and sensitivity analysis estimates for the effect per unit decrease in exposure-mediated natural log transformed C-reactive protein (CRP) levels on risk of the positive (rheumatoid arthritis) and negative (osteoarthritis) controls.

Proxied drug	MR analysis method	Rheumatoid arthritis Odds ratio (95% CI)	Osteoarthritis Odds ratio (95% CI)
Tocilizumab (*IL6R*)	IVW	**0.57 (0.39–0.82)**	0.82 (0.64–1.04)
	Weighted-median	**0.44 (0.32–0.61)**	0.83 (0.60–1.15)
	Weighted-mode	**0.51 (0.38–0.68)**	0.81 (0.60–1.09)
	MR-Egger	**0.28 (0.10–0.74)**	0.91 (0.47–1.75)
	MR-PRESSO	**0.53 (0.29–0.95)**	NA
	MR-RAPS	**0.51 (0.28–0.93)**	0.82 (0.64–1.04)
Canakinumab (*IL1β*)	IVW	**0.49 (0.30–0.79)**	1.23 (0.78–1.95)
	Weighted-median	**0.43 (0.23–0.81)**	1.30 (0.72–2.35)
	Weighted-mode	**0.24 (0.11–0.53)**	1.26 (0.70–2.29)
	MR-Egger	**0.13 (0.03–0.52)**	1.42 (0.35–5.72)
	MR-PRESSO	NA	NA
	MR-RAPS	**0.47 (0.29–0.75)**	1.24 (0.78–1.96)
Colchicine (β-tubulin gene family)	Wald Ratio	0.72 (0.02–21.51)	1.72 (0.10–28.08)
Overall CRP	IVW	**0.93 (0.87–0.98)**	0.95 (0.89–1.00)
	Weighted-median	0.96 (0.87–1.06)	0.96 (0.87–1.07)
	Weighted-mode	0.95 (0.86–1.04)	0.98 (0.88–1.08)
	MR-Egger	0.91 (0.88–1.00)	**1.13 (1.03–1.23)**
	MR-PRESSO	0.96 (0.91–1.02)	0.95 (0.90–1.00)
	MR-RAPS	0.94 (0.88–1.00)	0.96 (0.90–1.02)

Significant values are in bold.

NA for MR-PRESSO indicates that no outliers were detected by this method. *IVW* inverse variance weighted, *MR-RAPS* MR-Robust Adjusted Profile Score, *CI* confidence interval.

Similarly, the negative control demonstrated results as expected. There was no significant association between genetically proxied IL6R, IL1β or β-tubulin mediated lower CRP levels and risk of OA on primary or sensitivity analyses. There was also no robust significant association between genetically proxied reduced CRP and risk of OA (Table 1; Supplementary Tables 6–9).

The analysis for CAD demonstrated similar results to AS. Genetically proxied IL6R mediated lower CRP was significantly associated with lower risk of CAD on primary IVW (OR 0.66, 95% CI 0.54–0.80) and sensitivity analyses. There was no robust association between genetically proxied IL1β, β-tubulin or overall CRP and risk of CAD (Supplementary Tables 6–9).

## Discussion

By leveraging large scale GWAS data and the MR approach, we found that genetically proxied tocilizumab was associated with reduced risk of AS. There was no evidence of a statistically significant association between genetically predicted canakinumab and AS. Overall, the present study corroborates a potentially causal role of inflammation on AS, particularly through the IL6R pathway.

AS is known to share many risk factors with atherosclerosis. Current American and European guidelines recommend treatment of coexistent hypertension, and American guidelines additionally recommend statin therapy in patients with calcific AS and renin-angiotensin system blockade in patients who have undergone a transcatheter aortic valve implantation (TAVI)^3,4^. Several studies have demonstrated the benefits of immunomodulation in coronary artery disease, but no clinical trials have yet been performed among patients with AS. The Canakinumab Antiinflammatory Thrombosis Outcome Study (CANTOS) in 10,061 patients with previous myocardial infarction and elevated CRP demonstrated that IL1β inhibition with canakinumab was associated with reduced CRP levels and an associated reduction in cardiovascular events, independent of lipids and blood pressure^17,41^. Concordantly, the Cardiovascular Inflammation Reduction Trial in 4786 patients with previous myocardial infarction or multivessel coronary disease showed no reduction in IL1β, IL6 or CRP levels and correspondingly did not show any reduction in cardiovascular events among patients treated with methotrexate compared to placebo^42^. The Colchicine Cardiovascular Outcomes Trial (COLCOT) of 4745 patients with recent myocardial infarction demonstrated lower rates of further cardiovascular events in patients treated with colchicine compared to placebo, and the Low-Dose Colchicine trial in 5522 patients with chronic coronary disease similarly demonstrated reduced cardiovascular event rates in patients treated with colchicine compared to placebo^18,19^. While the present study did find a statistically significant association between genetically predicted colchicine and AS on Wald ratio analysis, the identification of only a single suitable SNP for analysis means that sensitivity analyses could not be performed. Notably, the identified SNP rs56283750 was identified as being in the promoter/enhancer region for the *TUBB3* gene, which is typically expressed in neuronal and testicular tissue, rather than cardiac tissue^43^. Moreover there are several isoforms within the β-tubulin family, and therefore genetic downregulation of one isoform may be compensated by upregulation of the others. If such compensation occurs, then a SNP in a given isoform would be unlikely to result in significantly altered peripheral CRP levels or clinically significant diseases. Additionally, the effects of colchicine are pleiotropic, incompletely understood and may not be adequately captured by reduction in CRP^44^. Further work is thus required to investigate the potential role for colchicine in AS.

There are no randomised trials of immunomodulatory medications in AS yet. Several observational studies have demonstrated an association between elevated inflammatory markers and AS. In a small study of 141 patients, elevated CRP was associated with AS, even after adjustment for traditional cardiovascular risk factors including hypertension and dyslipidemia^45^. In the larger Framingham Heart Study’s offspring cohort of 2683 participants, the group with aortic and/or mitral calcification had elevated levels of CRP and IL6, although this was no longer significant after adjustment for traditional cardiovascular risk factors^46^. In a histopathological study of 103 human aortic valves explanted at the time of aortic valve replacement, patients taking angiotensin receptor blockers had significantly lower fibrosis scores and IL6 expression but no difference in the amount of valve calcification^16^. In addition to blood pressure reduction, this anti-inflammatory and anti-fibrotic effect may be a contributing explanation to the previous observational findings that treatment with renin-angiotensin system blockade is associated with reduced mortality after TAVI^47,48^. A further histopathological investigation of 46 stenotic aortic valves explanted at the time of aortic valve replacement compared to 10 non-calcified controls with normal echocardiograms at the time of heart transplantation found that IL6 levels were nine times higher in the calcified compared to control valves^15^. A mouse model of AS induced by mechanical wire injury similarly demonstrated marked elevations in the inflammatory cytokines IL1β, IL6 and tumour necrosis factor α^49^. Consistent with this, an investigation of mice deficient in IL1 receptor antagonist demonstrated increased aortic valve leaflet thickness and transvalvular velocities compared to wild-type mice. This finding was independent of systolic blood pressure and lipids^50^.

The current conception of AS pathogenesis involves endothelial injury and dysfunction, lipid deposition, immune cell infiltration, osteogenic transition of valve interstitial cells and valve mineralisation^51,52^. This has important clinical implications, since treatments which target earlier stages may not be effective for late-stage disease and vice versa. IL6 is downstream of IL1β in the NF-*k*B pathway and upregulation of this pathway is thought to promote the osteogenic transition of interstitial cells that leads to valve mineralisation^52^. Our results suggest that immunomodulatory treatments that reduce IL6R signalling may therefore be expected to hinder AS at this stage in the pathogenesis. The neutral result from the IL1β analysis in the present study is notable given that IL1β is upstream of IL6R, and previously reported evidence supports a protective effect of canakinumab for patients with coronary disease. There are several possible explanations for this, including the smaller number of SNPs available for analysis for IL1β compared to IL6R. Furthermore, currently available data does not facilitate targeted MR analyses to distinguish factors which contribute to disease incidence versus progression, which would have important therapeutic implications for AS. This might be evaluated in future by GWAS analyses that are powered to evaluate cohorts with different AS severities. Additionally, the administration of immunomodulatory drugs is not without risk. The CANTOS trial, for example, demonstrated that canakinumab is associated with higher incidence of fatal infection compared to placebo^17^. Of note, this did not translate to a significant difference in all-cause mortality, and canakinumab importantly reduced the primary endpoint composite of nonfatal myocardial infarction, nonfatal stroke and cardiovascular death. Nevertheless, this does highlight the importance of considering the potential risks of immunomodulation along with the intended benefit.

The finding in the current study that overall CRP is associated with reduced risk of AS on IVW analysis should be interpreted cautiously given that the sensitivity analyses did not support a significant association, and there was evidence of significant heterogeneity and pleiotropy. Previous observational clinical studies have demonstrated associations between elevated CRP and AS^45,46^. Similarly lipoprotein(a), which is linked with pro-inflammatory effects, has been associated with increased risk of AS, and notably the relationship between lipoprotein(a) and AS is only modestly modulated by CRP levels^53^. Furthermore there is genetic evidence supporting a potentially causal relationship between lipoprotein(a) and AS^35^, yet MR studies evaluating CRP in diseases such as coronary artery disease have repeatedly shown no evidence of a causal role^54,55^. This is likely because CRP is a downstream inflammatory molecule. Hence CRP is expected to be elevated when upstream inflammatory processes are active, but CRP does not necessarily have a causal role in the pathogenic inflammation of coronary disease or AS itself. This would explain the observed elevation of CRP in AS and coronary disease, while also explaining the repeatedly negative results of MR analyses which aim to evaluate for a causal relationship between elevated CRP and coronary disease^54^. This is also the reason why it is reasonable to use CRP to identify relevant proxies for tocilizumab and canakinumab, as performed in the present study, since SNPs which reduce the function of *IL6R* and *IL1β* can be identified as those which reduce the levels of downstream molecules such as CRP. While an alternative methodological approach to the present study might have been to use GWAS analyses of peripheral IL6R and IL1β protein levels instead of CRP, there is currently a paucity of well-powered GWAS studies that analyse these proteins, with the available studies being at least an order of magnitude smaller than the CRP GWAS used in the present analysis. Moreover, the approach of using CRP as a downstream marker to identify genetic proxies has been identified as a suitable methodology in several previous studies^23–25^. Notably, however, whereas the analyses for *IL6R* and *IL1β* used CRP as a downstream marker to identify relevant genetic proxies, the analysis of overall CRP in the present study evaluated whether CRP itself has a causal role in AS. While the positive results from the primary IVW analysis suggest that CRP might have a causal role, there was notably significant heterogeneity identified in the IVW analysis, pleiotropy identified by the Egger intercept and the results of the weighted median, weighted mode and MR-Egger analyses were discordant with the primary IVW analysis. Taken together with previously published literature, it is less convincing that CRP itself has a pathogenic role in AS. Rather, the main utility of CRP appears to remain as a marker of active upstream inflammatory processes.

The present study investigates the potential effects of immunomodulation through drug proxies on AS. A strength of this analysis is that the MR results estimate the cumulative lifelong effect of genetically reduced inflammation. This is particularly relevant since inflammation is thought to occur early in the development of AS. Furthermore, since AS is a chronic condition with a long subclinical stage, a traditional randomised controlled trial will be costly and logistically challenging given the long follow-up time that will be required. However, it is unclear whether the results of the present study can be generalised to patients with established AS. It is possible, for example, that immunomodulation may be more effective, or even exclusively effective, at the initiation of AS pathogenesis.

The present study suggests that inflammation may have a causal role in the development of AS. Through genetic drug proxies, we demonstrate that inhibition of IL6R may reduce the risk of AS. In the absence of randomised trials, this study provides the most compelling evidence to date for the potential role of immunomodulation in the prevention of AS.

### Supplementary Information

Supplementary Figures.

Supplementary Tables.

## Data Availability

The data used in these analyses are publicly available. Summary statistics for CRP, RA and OA are available from: https://www.ebi.ac.uk/gwas/studies/GCST90029070, https://www.ebi.ac.uk/gwas/studies/GCST90013534 and https://www.ebi.ac.uk/gwas/studies/GCST005814, respectively. Summary statistics for AS are available from: https://doi.org/10.5281/zenodo.7505361, and for CAD from: http://www.cardiogramplusc4d.org/data-downloads/.
