# Supplementary material for: The effect of immunomodulatory drugs on aortic stenosis: a Mendelian randomisation analysis
Source: Sci Rep. 2023 Nov 1;13:18810. doi: 10.1038/s41598-023-44387-x (PMC10620428; doi:10.1038/s41598-023-44387-x)
Supplement: Supplementary file 1 — Supplementary Figures. [file 41598_2023_44387_MOESM1_ESM.docx]

**Supplementary Figures**

Tocilizumab

(Drug target: IL6R)

54 chromosomal regions (hg38) corresponding to gene, enhancers and promoters for *IL6R* identified using GeneHancer (Supplementary Table 1)

43,725 SNPs (db153) from these chromosomal regions identified using UCSC Table Browser

401 SNPs available in CRP GWAS^1^

43,324 SNPs excluded as they were not available in the CRP GWAS^1^

274 SNPs extracted that met the Bonferroni corrected p-value threshold for inclusion (0.05 / 43,725 )

18 SNPs available for harmonisation

127 SNPs excluded due to not being significantly associated with CRP

189 SNPs excluded due to LD with other variants or absence from the reference panel (r^2^ < 0.1, distance 1 Mb)

207 SNPs available from both the exposure and outcome datasets

67 SNPs excluded that were not available in the AS GWAS^2^ dataset

**A**

18 SNPs included for two-sample Mendelian randomisation (Supplementary Table 4)

0 palindromic SNPs excluded

Canakinumab

(Drug target: IL1β)

74 chromosomal regions (hg38) corresponding to gene, enhancers and promoters for *IL1β* identified using GeneHancer (Supplementary Table 2)

41,115 SNPs (db153) from these chromosomal regions identified using UCSC Table Browser

773 SNPs available in CRP GWAS^1^

40,342 SNPs excluded as they were not available in the CRP GWAS^1^

206 SNPs extracted that met the Bonferroni corrected p-value threshold for inclusion (0.05 / 41,115)

10 SNPs available for harmonisation

567 SNPs excluded due to not being significantly associated with CRP

143 SNPs excluded due to LD with other variants or absence from the reference panel (r^2^ < 0.1, distance 1 Mb)

153 SNPs available from both the exposure and outcome datasets

53 SNPs excluded that were not available in the AS GWAS^2^ dataset

**B**

9 SNPs included for two-sample Mendelian randomisation (Supplementary Table 4)

1 palindromic SNPs excluded

Colchicine

(Drug target: β-tubulin)

289 chromosomal regions (hg38) corresponding to gene, enhancers and promoters for *TUBB, TUBB2A, TUBB2B, TUBB3, TUBB4A, TUBB4B, TUBB6, TUBB1, TUBB8* and *TUBB8B* identified using GeneHancer (Supplementary Table 3)

179,171 SNPs (db153) from these chromosomal regions identified using UCSC Table Browser

1997 SNPs available in CRP GWAS^1^

177,174 SNPs excluded as they were not available in the CRP GWAS^1^

7 SNPs extracted that met the Bonferroni corrected p-value threshold for inclusion (0.05 / 179,171)

1990 SNPs excluded due to not being significantly associated with CRP

6 SNPs available from both the exposure and outcome datasets

1 SNPs excluded that were not available in the AS GWAS^2^ dataset

1 SNP available for harmonisation

5 SNPs excluded due to LD with other variants or absence from the reference panel (r^2^ < 0.1, distance 1 Mb)

**C**

1 SNP included for two-sample Mendelian randomisation (Supplementary Table 4)

0 palindromic SNPs excluded

C-reactive protein

47,998 SNPs that met p-value threshold for inclusion

(p < 5 x 10^-8^) in CRP GWAS^1^

34,021 SNPs available from both the exposure and outcome datasets

13,977 SNPs excluded that were not available in the AS GWAS dataset or for which allele nucleotides were not specified in the AS GWAS dataset^2^

887 SNPs available for harmonisation

33,134 SNPs excluded due to LD with other variants or absence from the reference panel (r^2^ < 0.1, distance 1 Mb)

**D**

886 SNPs included for two-sample Mendelian randomisation (Supplementary Table 4)

1 SNPs excluded due to incompatible alleles between exposure and outcome datasets or palindromic SNPs

Supplementary Figure 1: Flow chart of SNP selection process. **A.** SNP selection process to proxy tocilizumab (drug target IL6R) as the exposure and aortic stenosis (AS) for the outcome. **B.** SNP selection process to proxy canakinumab (drug target IL1β) as the exposure and AS for the outcome. **C.** SNP selection process to proxy colchicine (drug target β-tubulin) as the exposure and AS for the outcome. **D.** SNP selection process for overall CRP as the exposure and AS for the outcome.

**A**

**B**


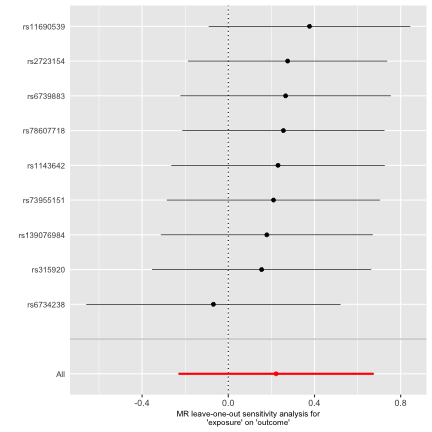

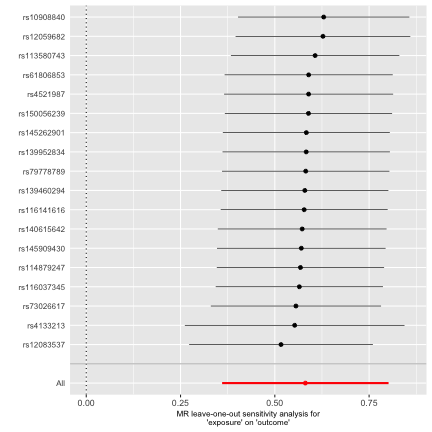


Supplementary Figure 2. Leave-one-out analyses for the associations between genetically predicted IL6R **(A)** and IL1β **(B)** mediated CRP modulation and aortic stenosis.

**A**

**B**

**C**


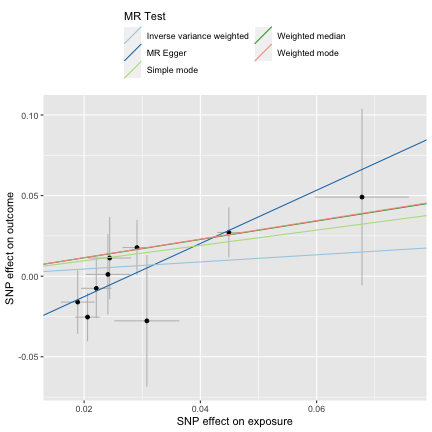

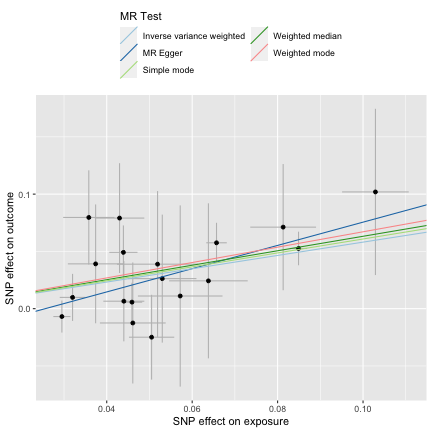

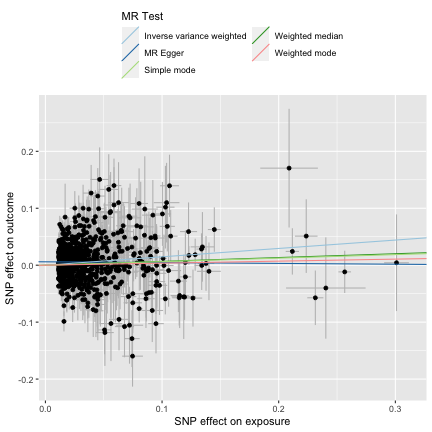


Supplementary Figure 3: Scatter plots for the associations between genetically predicted **(A)** IL6R mediated CRP modulation, **(B)** IL1β mediated CRP modulation and **(C)** overall CRP on aortic stenosis.

**A**

**B**

**C**


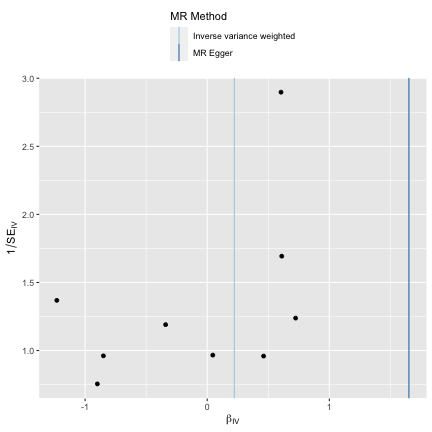

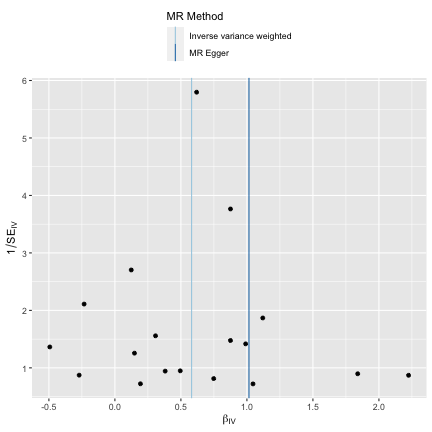

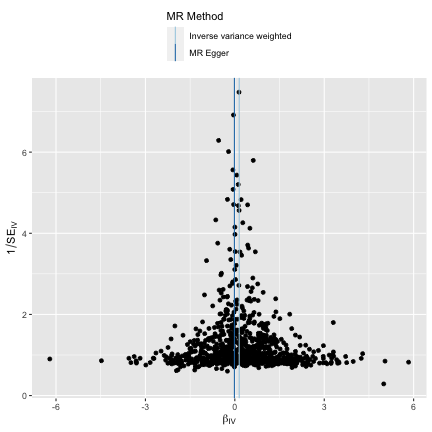


Supplementary Figure 4. Funnel plots for the associations between genetically predicted **(A)** IL6R mediated CRP modulation, **(B)** IL1β mediated CRP modulation and **(C)** overall CRP on aortic stenosis.


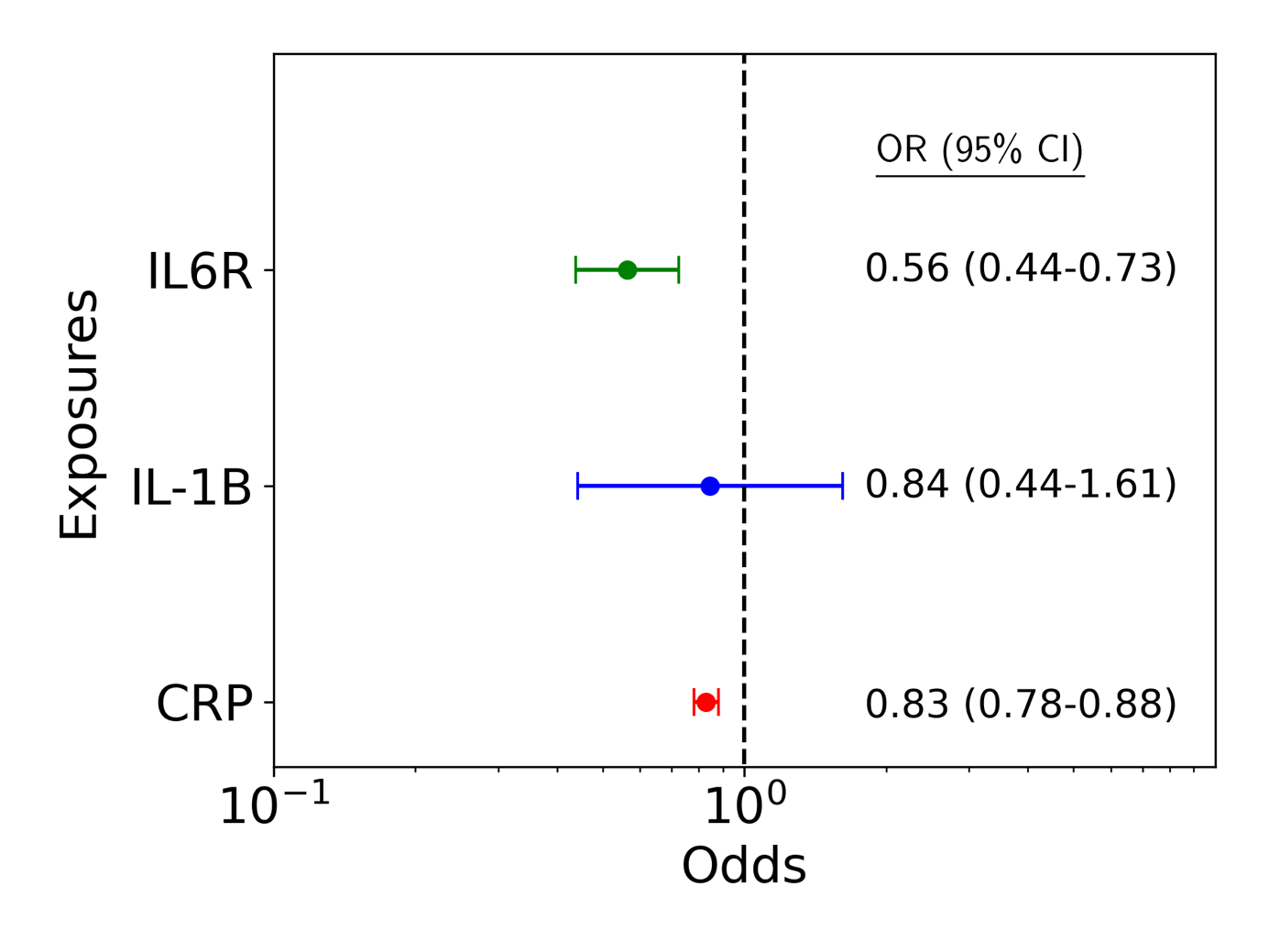


Supplementary Figure 5: Mendelian randomisation inverse variance weighted estimates for the effect per unit decrease in exposure-mediated natural log transformed C-reactive protein (CRP) levels on risk of aortic stenosis, with outliers excluded based on Cook’s distance. The exposures of interest included interleukin 6 receptor-mediated (IL6R), IL1β-mediated (IL1B) and overall genetically predicted CRP. OR: odds ratio; CI: confidence interval.


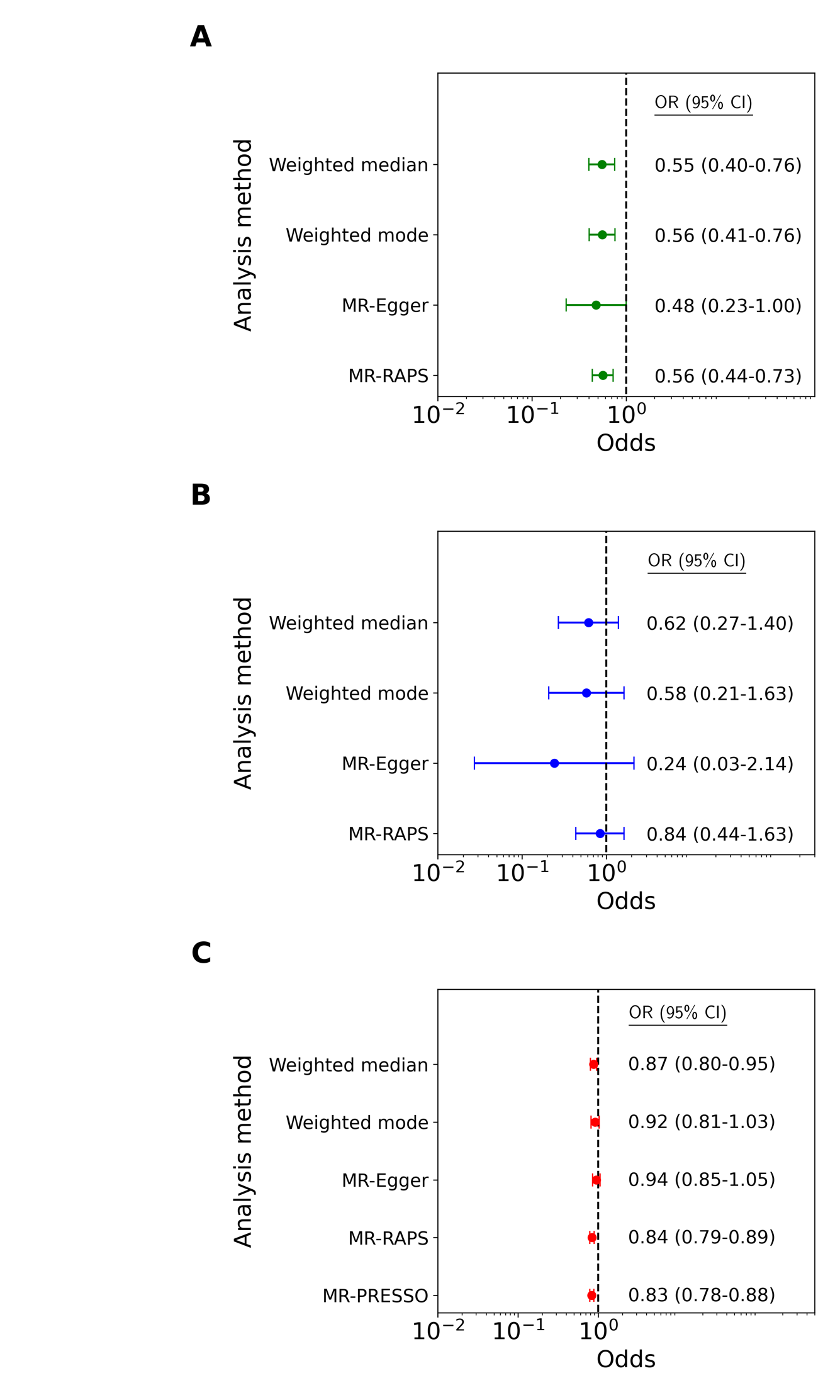


Supplementary Figure 6: Sensitivity analyses for the effect per unit decrease in exposure-mediated natural log transformed C-reactive protein (CRP ) levels on risk of aortic stenosis, with outliers excluded based on Cook’s distance. The exposures of interest included: **(A)** interleukin 6 receptor-mediated; **(B)** IL1β-mediated; and **(C)** overall genetically predicted CRP. OR: odds ratio; 95% CI: 95% confidence interval.

**A**

**B**

**C**


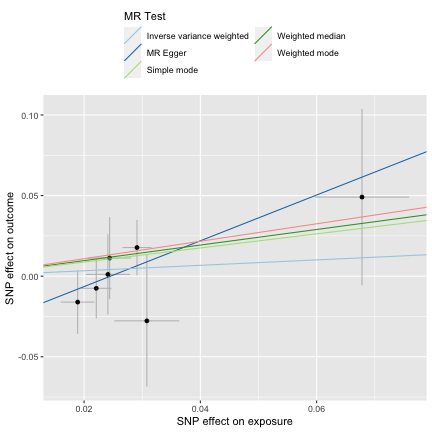

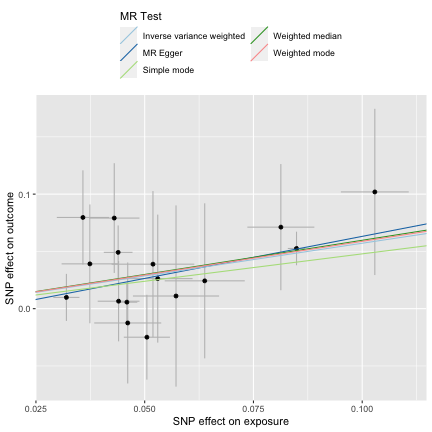

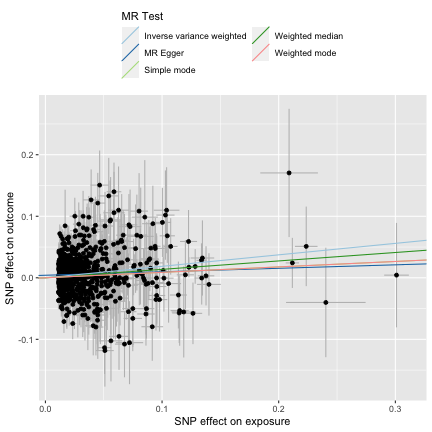


Supplementary Figure 7: Scatter plots for the associations between genetically predicted **(A)** IL6R mediated CRP modulation, **(B)** IL1β mediated CRP modulation and **(C)** overall CRP on aortic stenosis, with outliers excluded based on Cook’s distance.

**A**

**B**

**C**


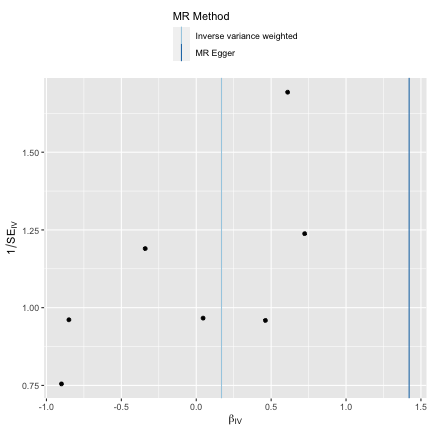

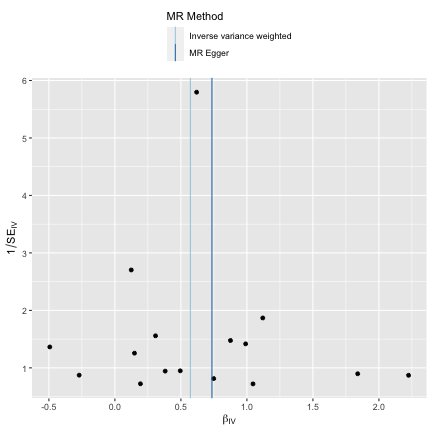

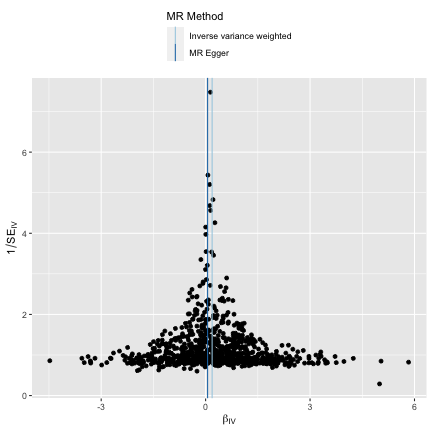


Supplementary Figure 8: Funnel plots for the associations between genetically predicted **(A)** IL6R mediated CRP modulation, **(B)** IL1β mediated CRP modulation and **(C)** overall CRP on aortic stenosis, with outliers excluded based on Cook’s distance.

References

1. Said S, Pazoki R, Karhunen V, et al. Genetic analysis of over half a million people characterises C-reactive protein loci. Nat Commun 2022;13(1):2198. DOI: 10.1038/s41467-022-29650-5.

2. Yu Chen H, Dina C, Small AM, et al. Dyslipidemia, inflammation, calcification, and adiposity in aortic stenosis: a genome-wide study. Eur Heart J 2023;44(21):1927-1939. DOI: 10.1093/eurheartj/ehad142.
